# Evaluation of Ozonation Technique for Pesticide Residue Removal in Okra and Green Chili Using GC-ECD and LC-MS/MS

**DOI:** 10.3390/plants11233202

**Published:** 2022-11-23

**Authors:** Susheel Singh, Vanrajsinh Solanki, Kirti Bardhan, Rohan Kansara, Trupti K. Vyas, Kelvin Gandhi, Darshan Dhakan, Hayssam M. Ali, Manzer H. Siddiqui

**Affiliations:** 1 Food Quality Testing Laboratory, N.M. College of Agriculture, Navsari Agricultural University, Navsari 396450, Gujarat, India; 2 Department of Basic Science and Humanities, Navsari Agricultural University, Navsari 396450, Gujarat, India; 3 Champalimaud Foundation, Lisbon, 1400038, Portugal; 4 Department of Botany and Microbiology, College of Science, King Saud University, Riyadh 11451, Saudi Arabia

**Keywords:** chili, acetamiprid, ethion, decontamination, dietary risk assessment, ozonated water

## Abstract

The indiscriminate use of pesticides in agricultural commodities has become a global health concern. Various household methods are employed to remove pesticide residues from agricultural commodities, e.g., water and ozone. Many ozone-based commercial pesticide removal machines are available in the market for the general public. The current study compares the pesticide removal efficiency of ozone-based washing of fruits and vegetables to simple tap water through commercially available machines and its health risk assessment to different age groups of consumers. The okra and green chili fruits were treated with acetamiprid and ethion as foliar application at the fruiting stage, using the recommended dose (RD) and double to the recommended dose (2RD), respectively. A modified QuEChERS-based pesticide extraction method was verified for its accuracy, precision, linearity, and sensitivity. The treated samples were washed with tap and ozonated water at different intervals, i.e., 3, 8, and 10 min using a commercial food purifier. Washing with ozonized water for 3 min recorded the maximum removal of acetamiprid and ethion from okra and chili fruits. Further, the risk quotient values (RQ) obtained were lower than one at both doses. Thus, washing vegetables with ozonized water for 3 min ensures vegetables are safer for general consumption without any health risk to Indian consumers.

## 1. Introduction

The excessive use of pesticides is a menace to human health and other biotas present in the ecosystem. Pesticides are man-made poisons, but when their residues or leftovers are detected in fruits and vegetables, they raise serious health concerns due to its acute and chronic adverse effects on human health. When entering the human body, many pesticides particularly organophosphate group pesticides including ethion, inhibit acetylcholinesterase (ACh), an enzyme that hydrolyzes acetylcholine at the synaptic junction [1]. Thus, pesticide residues in fruits and vegetables have chronic effects such as disrupting the endocrine system, cancer, respiratory illness, asthma, and diabetes [2,3]. Moreover, some pesticides are teratogenic, some cause congenital and neurological complications, and some cause people to suffer from reproductive abnormalities. Neonicotinoids and organophosphate pesticides are predominantly present in food commodities and are used throughout the world; these inhibit the ACh enzyme [1,4]. Acetamiprid is a neonicotinoid insecticide designed to target nicotinic acetylcholine receptors in insects, but its extensive use has led to adverse effects in non-targeted organisms including mammals. Prolonged environmental or accidental exposure to acetamiprid alters hematological, biochemical, and structural profiles, leading to neurological, hepatorenal, immunological, genotoxic, and reproductive effects [5]. Acetamiprid and ethion residue accumulation in arable soils and soil water, and runoff into waterbodies could pose a severe threat to contamination of environmental matrices [6,7]. Ethion, an organophosphate, and acetamiprid, a neonicotinoid, are profusely used on green chili (to control mites and thrips) and okra (most effective against Jassids), respectively [8]. In India, a fatal poisoning accident occurred when nine adults and six children died within 24 h of consuming ethion-tainted food [9]. Due to indiscriminate and injudicious pesticide use, food items, mainly fruits and vegetables, are frequently found to contain dangerous levels of pesticides, posing unexpected scenarios for consumers. Pesticide residues such as acetamiprid and ethion have occasionally been identified in okra and green chili at levels exceeding the maximum residue limits (MRLs) due to injudicious use and non-compliance with postharvest intervals [10].

For this study, Okra (*Abelmoschus esculentus* L. Moench) and green chili (*Capsicum annuum* L.) were used which are one of the important commercial vegetable crops grown in India, producing 3,737,000 million tons of green chili from an area of 0.36 million ha and 6,219,000 million tons of okra from 0.5 million ha during the year 2018–19 [11]. Owing to its natural taste, nutritional quality and diverse cooking methods, these vegetables are the major components of a vegetarian diet in India [12]. Even the European Union Commission (EU) as well as the Saudi Food and Drug Authority (SFDA) added okra and green chili in high-risk categories due to exceeding levels of pesticide residues in vegetables consignments exported from India [13]. Therefore, there is a need to develop methodologies to dislodge pesticide residues from okra and chili fruits to ensure safety and a healthy way to consume fresh produces.

Some strategies should be identified to reduce pesticide residues in food commodities. Among them, such methods used to decontaminate the foods are washing with water, soaking in salt or some chemical solutions (such as chlorine, chlorine dioxide, hydrogen peroxide, ozone, acetic acid, hydroxy peracetic acid, iprodione, and detergents), and hypochlorite salts. The use of hypochlorite salt has drawbacks since it is unstable and releases poisonous chloride, which causes chemical pollution [14], so there is a great urge to find alternative sound methods [15]. Recently, one such method where ozone was used to remove pesticide residues from fruits and vegetables had several advantages: it was economic, efficient, easy to operate, it preserved the food qualities, and was relatively safe [16]. Ozone (O_3_) is one of the most potent sanitizers used against a wide spectrum of microorganisms (reduce toxicity of mycotoxins) as it is a strong oxidant (2.07 mV) [17,18,19]. In 1840, Christian Friedrich Schönbein discovered ozone in Germany and it was first used commercially in France to treat potable water. In the food processing industry, ozone has recently been recognized as a novel emerging non-thermal technology for the degradation of pesticide residues from fresh produce. It is also regarded as a green technology because, unlike the other traditional methods, ozone treatment leaves little trace in foods [20]. The solubility of ozone in water is 1.5 mg L^−1^ at 30 °C and it has a half-life ranging from 20 to 30 min in distilled water at 20 °C. Moreover, ozone falls under the GRAS (Generally Recognized As Safe) category and is identified as a strong disinfectant and oxidizing agent to reduce water-soluble pesticides [14,21]. Thus, the application of ozonated water emerged as a promising technique for removing pesticide residues from vegetables and fruits [21,22,23]. In order to remove pesticide residues from vegetables, Chen et al. (2007) developed a novel domestic-scale machine that includes a closed cleaning chamber, an ozone generator, a water recirculation pump, and an oxidation-reduction potential (ORP) electrode [24]. Ozone has a slow rate of reaction and does not always completely oxidize all organic compounds. In order to effectively detoxify industrial effluents, pharmaceuticals, pesticides, and recalcitrant organics, advanced oxidation processes based on ozone, such as O_3_/UV, O_3_/H_2_O_2_, O_3_/Fe(II), O_3_/metal oxide catalyst, O_3_/activated carbon, and O_3_/Fenton, have recently undergone scientific evaluation [25]. In the present experiment, a commercial fully automatic ozone food purifier with a vortex ozone system having capacity of water 8 L, ozone output of 200 mg/h, pressure 0.50 kg/cm^2^ was used. It consisted of three operation modes of 3, 8, and 10 min. The treated samples were washed separately with tap water (having pH = 7.65, TDS = 103 ppm and EC = 1454 µS) and ozonized water through a commercially available ozone food purifier at three different time intervals.

Hence, the present study aims to evaluate the pesticide removal efficiency of ozonated and tap water rinsing of okra and green chili treated with acetamiprid and ethion through a commercially available ozone-based food purifier machine and its risk assessment study on human health.

## 2. Results

### 2.1. Verification of Method Performance for Insecticide Extraction

**Figure 1:**
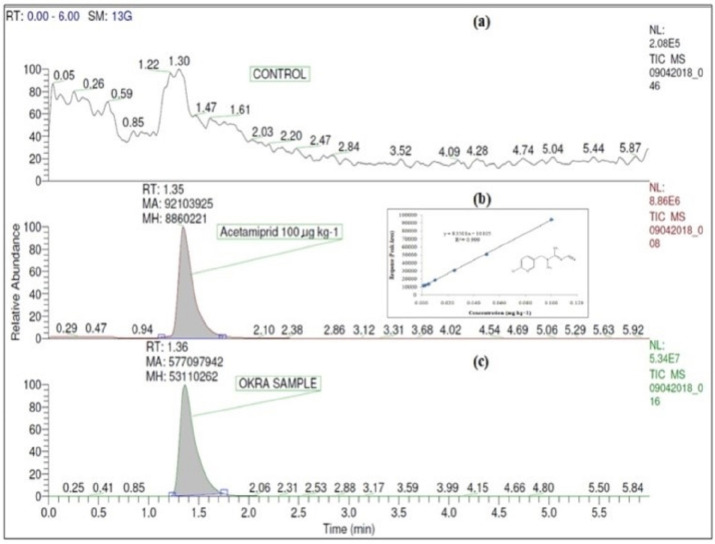
UHPLC-MS/MS Selected Reaction Monitoring (SRM) chromatogram of acetamiprid (**a**) in okra matrix-sample blank, (**b**) at standard 0.1 mg/kg with linearity, and (**c**) in okra sample (unwashed, treated, W0T0).

**Figure 2:**
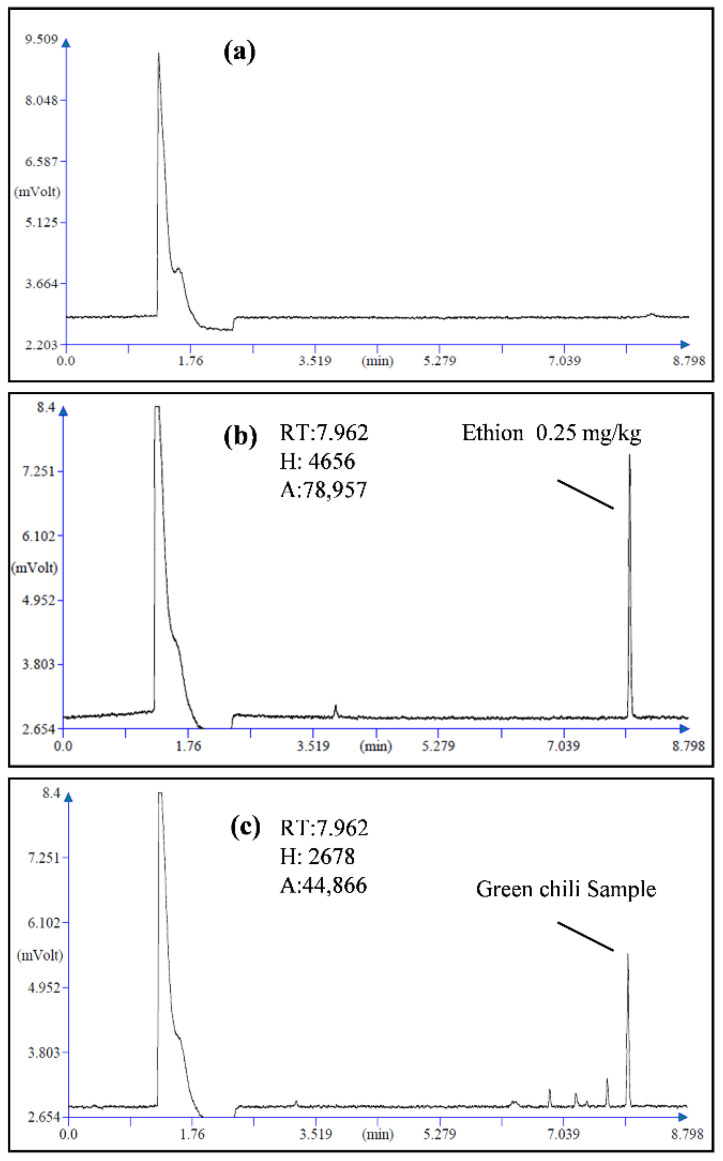
Optimized GC-ECD chromatogram of ethion (**a**) in green chili matrix- sample blank, (**b**) at standard 0.25 mg/kg with linearity, and (**c**) in green chili okra sample (unwashed, treated, W0T0).

The verification of the pesticide extraction and detection method was performed by determining the linearity, LOD, LOQ, accuracy, and precision of the analytical method [26]. After injecting working standard solutions of acetamiprid (0.1 mg/kg) into the UHPLC and ethion (0.25 mg/kg) into the GC, the obtained retention time (RT) was at 1.35 and 7.96 min, respectively (Figure 1b or Figure 2b). The MRLs for pesticides in food matrices are considered to be the upper limit of the analytical method to determine the suitability for determining pesticide residue contents. A MRL is defined as the highest level of a pesticide residue that is legally tolerated in or on food or feed when pesticides are applied correctly in accordance with Good Agricultural Practice [27]. The LODs and LOQs of the pesticides were determined using the signal-to-noise ratio. Thus, the LOD and LOQ of acetamiprid were found to be 0.002 and 0.007 mg/kg, respectively, while those of ethion were 0.006 and 0.018 mg/kg, respectively (Table 1). The MRLs of acetamiprid and ethion in okra and green chili are 0.2 and 5.0 mg/kg, respectively [27], which are much higher than the respective LOQ values for acetamiprid and ethion. Hence, the analytical method fulfills this criterion. The linear relationships among the ratios of the peak area and the corresponding concentrations were obtained. The regression equation for acetamiprid was y = 83501x + 10105 and that for ethion was y = 16372x + 34.75. Their correlation coefficients (R^2^) were 0.999 for both insecticides (Table 1). The mean recoveries and the relative standard deviations (RSDs) of acetamiprid ranged from 89.13 to 104.05% and from 13.41 to 16.65% in okra, and in the case of ethion, from 85.82 to 88.47% and from 11.94 to 16.57% were noted in green chili (Table 1). Thus, the average recovery rate of acetamiprid was higher than that of ethion. All the results of recoveries were within the acceptable limit from 70 to 120% and the RSD was ≤20 [26]. Consequently, these studies sufficiently verified that the analytical method adopted for the extraction of acetamiprid and ethion from okra and green chili satisfy the acceptance criteria of the method performance [26].

### 2.2. Pesticide Removal Efficiency

#### 2.2.1. Acetamiprid in Okra

**Figure 3:**
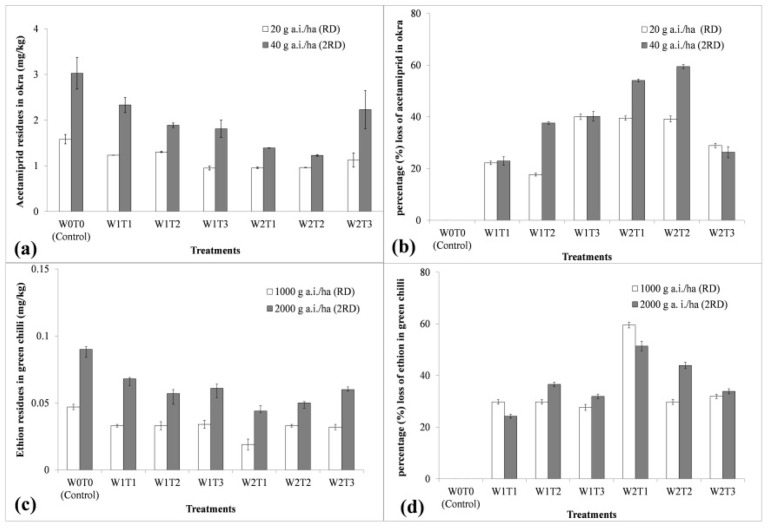
Decontamination of pesticide residues in vegetables: (**a**) acetamiprid residues (mg/kg), (**b**) percentage loss of acetamiprid residues in okra sample, (**c**) ethion residues (mg/kg), and (**d**) percentage loss of ethion residues in green chili sample (*n* = 3). [W0T0: treated unwashed control (non-ozonated); W1T1: washing with tap water for 3 min; W1T2: washing with tap water for 8 min; W1T3: washing with tap water for 10 min; W2T1: washing with ozonized water for 3 min; W2T2: washing with ozonized water for 8 min; W2T3: washing with ozonized water for 10 min; RD: recommended dose; 2RD: double to the recommended dose; mean ± Standard deviation (SD); percentage loss of acetamiprid and ethion residues over initial concentration recorded in control].

The residues of acetamiprid in okra at RD and 2RD were detected at 1.582 and 3.026 mg/kg, respectively, in the control (W0T0) (Figure 1c and Figure 3a). The reduction in the concentration of acetamiprid due to the tap and ozonated water washing in okra were from 17.70 to 37.61% and from 39.19 to 59.45% at RD and 2RD, respectively (Figure 3b). Significantly, the lowest concentration of acetamiprid residues, 0.949 mg/kg at RD, was recorded with the treatment receiving tap water washing for 10 min (W1T3). Statistically, it remained at par with the treatments W2T1-ozonized water washing for 3 min (0.957 mg/kg), W2T2-ozonized water washing for 8 min (0.962 mg/kg), and W2T3-ozonized water washing for 10 min (1.125 mg/kg). The maximum percentage loss of acetamiprid residues was 40.01% in the treatment W1T3 over the initial concentration in control (W0T0) at RD. However, in the case of 2RD, the treatment W2T2 exerted the lowest acetamiprid concentration (1.227 mg/kg), which was at par with the treatments W2T1 (1.227 mg/kg) and W1T3 (1.810 mg/kg) (Figure 3a). The data obtained in our study signified that the rinsing of okra fruits (treated with acetamiprid) with ozonated water washing for 3 min recorded a higher reduction in the chemical concentration at RD and 2RD. The current study’s findings are close to those of other researchers who had tested the acetamiprid decontamination capacity of ozonated water from okra and strawberries [22,28].

#### 2.2.2. Ethion in Green Chili

The residues of ethion recorded in green chili for the control (W0T0) were 0.047 and 0.090 mg/kg at RD and 2RD (Figure 2c and Figure 3c). The reduction in the concentration of ethion due to tap and ozonated water washing recorded in green chili were from 27.66% to 59.57% and from 24.23 to 51.41% at RD and 2RD, respectively (Figure 3d). The maximum reduction in concentration over the initial loading of ethion residues in green chili was recorded 0.019 mg/kg for treatment W2T1-ozonized water washing for 3 min at RD and 0.044 mg/kg at 2RD (Figure 3c). Moreover, the maximum percentage loss of acetamiprid residues were 51.41% and 59.57% at RD and 2RD. However, the treatment W1T2 (0.057 mg/kg) and W2T2 (0.050 mg/kg) were at par with treatment W2T1 at 2RD (Figure 3c,d). Thus, washing with ozonized water for 3 min (W2T1) recorded the highest significant reduction in the concentration of ethion at RD and 2RD for green chili. Thus, these observations indicated that both cleaning methods can reduce pesticide residues in the vegetables to a certain extent; however, the ozonized water treatment with a particular time duration (3 min) resulted in a better treatment effect than non-ozonized water. Figure 3 showed that pesticide residues are lower when washing with ozonized water for 3 min (W2T1) for both commodities. The advantageous ozone application phenomenon would be explained as in discussion.

### 2.3. Dietary Risk Assessment

The pooled residue data obtained from okra and green chili were used for calculating the risk assessment parameters. Although the RQ values recorded across the treatments and over the doses reflect that these values are generally lower than 1, it is also observed that the RQ values of the treatments with ozonized water washing are comparatively lower than tap water washing (Table 2 and Table 3). However, the RQ values recorded for treatment (W2T1), i.e., ozonated water washing for 3 min, were the lowest among other treatments in both commodities at the recommended dose (RD). It indicates that the water washing drastically reduces the pesticide residue load, but washing with ozonated water shows a higher efficiency in lowering the pesticides at RD. Therefore, the consumption of okra and green chili laced with acetamiprid and ethion subjected to washing with ozonated water for 3 min is safer. Their RQ values are much lower than recommended [29]. 

## 3. Discussion

To mitigate the demand of a growing population and to prevent economic loss, the application of pesticides is necessary for fruit and vegetable crops. However, excess pesticide applications result in pesticide residues and its metabolites in fruits and vegetables that have a detrimental effect on human health. They are not only hazardous to human health, but they may also cause serious damage to soil ecosystems, water bodies, and soil and water biodiversity [30]. Various techniques are used to remove pesticides from agricultural produce. Among them, one such promising technique is washing with ozonated water. Due to its feasibility, one can even use it at home to remove pesticide contamination from fruits and vegetables.

An advantage of the application of ozone is that it can be generated at the site of use and it can be applied in gaseous form. The most significant characteristic of O_3_ is its prompt decomposition ability into simple oxygen. Thus, there are no such safety concerns about the consumption of residual by-products and they do not cause secondary pollution [14]. Numerous studies have shown that ozone can deplete or eliminate pesticide residues from the surface of fruits and vegetables and their results are in accordance with present findings [22,31,32,33,34,35]. The present study revealed that ozonated water was more effective than tap water in the removal of pesticides. Additionally, removal efficiency increased when vegetables were treated with ozone and the hypothesis could also be confirmed through other experimental results [24]. Another possibility of variation in the content of the different compounds’ reductions is also served by their molecular weight. The reduction is possible because the dissolved ozone generates hydroxyl radicals that are highly effective in decomposing organic molecules such as pesticide residues [36,37] and washing with ozone water was more effective in the removal of pesticides with lower molecular masses (acetamiprid 222.67 g/mol and ethion 384.48 g/mol). A similar finding was also observed during the removal of 16 pesticide residues from strawberries using ozonated water compared to washing with tap water [22]. Thus, it can also be concluded that the molecular weight of each pesticide could also affect the percentage of reduction. However, it has been observed that increments in the contact period of washing either with ozonated water or tap water increased the residues of pesticides. This might be due to the re-absorption of pesticide residues from water/ozonated water back to commodities. This finding is reflected by how washing treatments with high contact periods show similar efficiency with higher pesticide contents (Figure 3).

The acetamiprid (log K_ow_ = 0.8 at pH 7, 20 °C; [38]) pesticide penetrated deep inside the okra skin, which is reflected by the lower removal rate. However, non-systemic acaricide ethion (log K_ow_ = 5.07 at pH 7. 20 °C) was more easily removed from green chili [38,39]. Despite acetamiprid presenting higher solubility in water (4.25 × 10^3^ mg/L at 25 °C) than ethion (2.0 mg/L), it was not the pesticide that was mostly removed by the different washing processes. This might be due to the complex composition of the okra matrix over green chili. Green chili fruits are covered by a thick coating of a cuticle layer composed of fatty acids that limit water loss from the fruit [40]. Thus, ethion is restricted to penetrating into the green chili. Considering the consistency of pesticide removing ability over two years, the data revealed that treatment W2T1, i.e., washing with ozonized water for 3 min, is more efficient in the removal of pesticide acetamiprid and ethion residues from okra and chili, respectively.

The rationale behind the removal/decontamination of insecticides is their physicochemical properties and their interaction with different matrices. One such important physicochemical property is the Octanol/water partition coefficient (K_ow_) parameter, which indicates insecticide’s ability to mix with polar and nonpolar media. The log K_ow_ values are generally inversely related to aqueous solubility. The pesticides with high log K_ow_ values can be quickly absorbed and strongly retained by waxes on the skin of commodities such as fruits and vegetable and, once retained by the cuticle wax layer, the pesticides are not easily removed by washing. The first hypothesis about the removal order of pesticides is the influence of their octanol/water partition coefficient (K_ow_ = concentration in Octanol/concentration in water) or their solubility [39].

The features of the pesticide, such as its overall stability as a parent molecule or as metabolites, its volatility, solubility, formulation, and the mode and site of application, are important factors that affect the persistence of pesticides in plants. Environmental factors, notably those related to temperature, precipitation, humidity, and air movement, however, also have a substantial impact on pesticide persistence. The physical properties of the pesticide and its chemical stability appear to be the most crucial of these aspects; otherwise, the solubility of the pesticide in plants would be more significant. The species and pace of growth of a plant appear to be the most significant of its traits and have the biggest impact on the pesticides’ abilities to remain in the plants [41]. In the present experiment, the insecticides were sprayed at the physiological maturity stage of okra and green chili, so that the pesticide removal efficiency could be evaluated as fruits of okra and green chili are harvested at this stage and available for general consumption. Thus, the application of insecticide at the physiological maturity stage of okra and green chili is the right stage to check the removal efficiency of ozonated and tap water from these commodities. As mentioned above, the plant’s genetic characteristics and growth habits have a pronounced impact on pesticide metabolism but are out of the scope of the current study; they were thus not considered in the present study. However, consideration of these factors along with the physico-chemical properties of pesticides and environmental factors could help to determine the pesticidal persistence and dislodge behavior in plants. Moreover, the effectiveness of the commercial ozone-based food purifier with the vortex ozone system should further be evaluated on different crops and different classes of pesticides with varied time periods of washing 

## 4. Materials and Methods

### 4.1. Field Experiment

The experiment was conducted on green chili (variety *Chilli-111*) and okra (var. *Gujrat Anand Okra-5*) at the Horticulture Polytechnic farm, Navsari Agricultural University (NAU), Navsari, Gujarat, India, with good agricultural practices (GAP). The farm is located at 20°92′ N and 72°89′ E at an altitude of about 10 m above MSL (mean sea level). The raised plants were subjected to the foliar application of acetamiprid (Reelik^®^20% SP) on okra and ethion (Rusmite^®^50% EC) on the chili at the fruiting stage. The experiment was conducted in a randomized complete block design with three replications along with one untreated control (spray of water). The treatments applied were recommended doses (RD) of 20 g a.i./ha and double the recommended dose (2RD) at 40 g a.i. ha^−1^ on okra and the recommended dose (RD) 1000 g a.i. ha^−1^ and double to the recommended dose (2RD) 2000 g a.i./ha on the green chili. The rate of application of insecticide were adopted as per the recommendation of Central Insecticide Board and Registration Committee; the apex statuary body of Government of India responsible for regulating the use of pesticide in India [42]. The data were recorded for two years. Furthermore, the pooled values obtained during the two-year (2017–18 and 2018–19) studies were considered for statistical analysis and the significance was measured by the Least Significance Difference at *p* = 0.05.

### 4.2. Sample Collection and Ozonation Treatment

One kg okra and green chili samples were collected from each treated plot and later composited. The composited samples were sealed packs in clear, transparent, and autoclavable disposable bags (HiDispo^TM^ Bag-74; Hi-media; Size: 34″ × 20″), labeled, quickly refrigerated, and transported to the laboratory in an icebox to retain their freshness.

For ozonation purposes, Prism Agritech Solution manufactured commercial Safeozone^TM^-fully automatic ozone food purifier with vortex ozone system with an 8 L capacity of water, ozone output of 200 mg/h, and pressure 0.50 kg/cm^2^ was purchased and used. It consisted of three operation modes of 3, 8, and 10 min. For all experiments the operating conditions were room temperature and pressure. The treated samples were washed separately with tap water (having pH = 7.65, TDS = 103 ppm and EC = 1454 µS) and ozonized water through a commercially available ozone food purifier at three different time intervals. Two parameters, i.e., the washing process (W1—Washing with tap water; W2—Washing with ozonated water), and time (T1—3 min, T2—8 min, and T3—10 min) were used to treat the samples. Thus, a total of six treatments were used, i.e., W1T1: washing with tap water for 3 min; W1T2: washing with tap water for 8 min; W1T3: washing with tap water for 10 min; W2T1: washing with ozonized water for 3 min; W2T2: washing with ozonized water for 8 min; and W2T3: washing with ozonized water for 10 min. Moreover, pesticides treated one sample for both doses was kept as such separately and analyzed for respective insecticides in okra and green chili (W0T0). Furthermore, sample blanks consisting of both crop matrices were also checked on the respective instruments to determine any interference (Figure 1a and Figure 2a).

### 4.3. Sample Extraction and Cleanup

The samples were processed and analyzed in the Department of Pesticide Residue, Food Quality Testing Laboratory, NAU, Navsari, Gujarat, India. Each sample was pre-treated as per the modified QuEChERS (Quick, Easy, Cheap, Effective, Rugged, and Safe) method for fruits and vegetables [43,44] before pesticide residue detection. After washing with ozone and tap water, the okra and green chill fruit samples were cut and homogenized by a heavy-duty homogenizer and a representative sample (15 ± 0.1 g) was taken in 50 mL capacity polypropylene tubes. To this, 15 mL of 1% acetic acid in acetonitrile was added into polypropylene tubes and kept in a deep freeze for 20–30 min incubation. After incubation, 6.0 g of MgSO_4_ and 1.5 g of sodium acetate were added to the tube, vortexed for 1.0 min, and the samples were centrifuged for 2.0 min at 2205× *g*. The supernatant (6.0 mL) was transferred in 15 mL capacity polypropylene tubes containing a mixture of 0.9 g of MgSO4 and 0.3 g of PSA (primary secondary amine), vortexed for 1.0 min, and then centrifuged 2.0 min again at 1125× *g*. From the supernatant, 2.0 mL was taken into 15 mL capacity test tubes and evaporated to dryness with nitrogen gas using low volume evaporator (TurboVap^®^) until it was almost dry. After evaporation, the acetamiprid residues were reconstituted to 2.0 mL with methanol: water (80:20; *v*/*v*), while the ethion residues were in n-hexane: acetone (9:1; *v*/*v*) for chromatographic analysis. Before being injected on respective instruments, the samples were filtered through syringe filters (0.02 µm, pore size). The GC-ECD analysis, which is appropriate for the analysis of non-polar compounds that can withstand higher temperatures, was applied for ethion, whereas LC-MS/MS, which is better suited for polar compounds and heat labile chemicals, was used for acetamiprid determination.

### 4.4. Verification of Method Performance for Insecticide Extraction

The performance of the method was developed and validated as per SANTE guidelines [26] by studying different parameters that include linearity, the limit of detection (LOD), and the limit of quantification (LOQ), accuracy, and precision. The linearity of acetamiprid and ethion were obtained using six calibration standards ranging from 0.001–0.1 mg/kg and 0.025–1.0 mg/kg, respectively. The LOD for both the analytes was calculated as LOD (mg/kg) = (mean of standard deviation/Slope) × 3, while the LOQ of both analytes was calculated as LOQ (mg/kg) = (mean of standard deviation/Slope) × 10 [45]. The accuracy and precision were evaluated through a recovery study for the pesticides. Three concentration levels of fortification for acetamiprid (0.025, 0.050, and 0.100 mg/kg) in the okra and ethion (0.100, 0.250 and 0.500 mg/kg) in green chili were used with seven replications. The consistency of the recovery study result reflects the precision, which can be represented by the relative standard deviation (RSD%). The percentage of pesticides removed was calculated using the equation: pesticide residue (%) = (concentration of pesticide residues after vegetable treatment with ozonated water or tap water-initial concentration of pesticide residues in untreated vegetable W0T0)/(Initial concentration of pesticide residues in untreated vegetables W0T0) × 100.

### 4.5. Acetamiprid Detection in Okra by LC-MS/MS Analysis

The quantitative analysis of acetamiprid was performed on a TSQ Quantum Access MAX triple stage quadrupole mass spectrometer (Thermo Scientific, Waltham, MA, USA) with a heated electrospray ionization (HESI) source. A Dionex-made ultra-high performance liquid chromatography (UHPLC) system (model: Dionex Ultimate 3000 RS) equipped with an autosampler, a quaternary pump system, and a column compartment was used for acetamiprid. The separation was achieved on the Hypersil Gold C18 column (150 × 4.6 mm, 5 μm particle size) with a 0.3 mL/min flow rate at 30 °C. An elution gradient was used with solvents, A: water with 5mM ammonium formate, 0.1% formic acid, and B: methanol with 5mM ammonium formate, 0.1% formic acid: with gradient profile (*t* (min), %A): (0, 98), (0.5, 98), (2, 60), (5, 98), and (6, 98). The mass spectrometry parameters were optimized in positive ionization mode (ESI^+^) with capillary voltage 4500 V; vaporizer temperature 350 °C; sheath gas (N_2_) 48 arbitrary units; aux gas (N_2_) 18 arbitrary units, and ion transfer capillary temperature 325 °C. The selective reaction monitoring (SRM) mode was adopted during this study. The masses of acetamiprid were monitored and optimized using standard parameters: precursor ion 222.94 *m/z* and product ions 126.00 *m*/*z* (collision energy: 22 eV) and 127.19 *m*/*z* (collision energy: 21 eV). The data were processed using the LCQUANTM 2.9 QF1 software (Thermo Scientific).

### 4.6. Ethion Detection in Green Chilli by GC-ECD

A gas chromatograph (Trace GC Ultra^®^) equipped with the electron capture detector (ECD) and TRIPLUS auto-sampler (AI 1310) was used for the quantitative analysis of ethion from green chili. The chromatographic separation was performed on a capillary column (AB-5, 30 m × 0.25 mm i.d.× 0.25 µm FT, Thermo Fisher, USA). The specific conditions were as: the 1.0 µL sample was injected under splitless mode into GC with ultra-pure helium (99.999 %) gas as carrier gas at a flow rate of 1.0 mL/min, while the oven temperature was initially maintained at 160 °C for 1 min and programmed with the ramp of 15 °C/min to attain the final temperature of 300 °C, which was maintained for 3 min; the injector and detector temperatures were maintained at 230 and 300 °C, respectively. The reference current of ECD was 1.0 nA. The data were processed using the Xcalibur software (Thermo Scientific).

### 4.7. Dietary Risk Assessment

The estimated daily intake (EDI) of acetamiprid and ethion residue was calculated by multiplying the average residues (mg/kg) (pooled values) obtained from washing with tap and ozonized water with the average food consumption rate (g/day) divided by the mean weight of a different group of Indian consumers (kg) [46]. The long-term risk assessment of intakes compared to pesticide toxicological data were assessed by calculating the risk quotient (RQ), dividing the EDI by the relevant acceptable daily intake (ADI) expressed in mg/kg body weight (bw) day^−1^. The ADI values of acetamiprid and ethion are 0.07 and 0.02 mg/kg bw/day [47]. The acceptable risk for long-term human dietary intake of pesticides is confirmed when the RQ is less than 1 and if the RQ is more than 1 it is an unacceptable risk [48].

### 4.8. Data Analysis

The data were statistically analyzed for the effect of water washing and ozonized water washing of chili and okra. The pesticide residue data were analyzed using a completely randomized design (CRD) and the CD, CV, and SEM values were calculated [49].

## 5. Conclusions

The rinsing of pesticide-laced matrices with tap water is a conventional technique while using ozonated water is an emerging technique for the removal of pesticide residues. Hence, a comparative study was conducted to determine the pesticide removal from the matrices through washing with ozonated and tap water. The novel finding of the present investigation enables us to determine the superiority of ozone-based water rinsing over tap water to minimize the load of toxic pesticide residues from vegetables, i.e., okra and green chili. Furthermore, the risk-reduction capacity of ozonated water washing for various pesticides in vegetables and fruits was evaluated for Indian consumers. The present study revealed that ozonation treatment effectively removes acetamiprid and ethion residues in okra and green chili fruits by washing for 3, 8, and 10 min. There was a significant reduction in both insecticides when washed with ozonized water for 3 min in okra and green chili. Hence, homemakers, consumers, and food processors can use 3 min to wash with a commercially available Vortex Ozone Technology (with an ozone-producing capacity of 0.5 kg/hour ozone) to remove acetamiprid and ethion residues from chili and okra. Thus, pesticide degradation using ozone will help people to judge the status of pesticides in vegetables as well as render a base for controlling agricultural pesticide residues during the household environment. In addition, the finding of this study is useful to save the consumers’ health and also equally helpful to sustain the economic interest of industrial fruits and vegetable exporters.

## Figures and Tables

**Table 1:** Method performance verification studies of acetamiprid in okra and ethion in green chili.

Parameters	Particular	Acetamiprid		Ethion	
Linearity (*n* = 5)	Calibration concentration range	0.001–0.1 mg/kg		0.005–1.0 mg/kg	
	Regression equation	y = 83501x + 10105		y = 16.372x + 34.75	
	R^2^ [R^2^ ≥ 0.99]	0.999		0.999	
Sensitivity (*n* = 5)	LOD (mg/kg)	0.002		0.006	
	LOQ (mg/kg)	0.007		0.018	
Accuracy (*n* = 7)	Percentage Recovery [70–120%]	Fortified level (mg/kg)	(%)	Fortified level (mg/kg)	(%)
		0.025	95.80 ± 4.44	0.100	85.82 ± 6.16
		0.050	89.13 ± 6.78	0.250	88.47 ± 7.89
		0.100	104.05 ± 6.26	0.500	86.52 ± 7.96
Precision (*n* = 7)	RSD [≤ 20%]	Fortified level (mg/kg)	(%)	Fortified level (mg/kg)	(%)
		0.025	15.99	0.100	16.53
		0.050	16.65	0.250	11.94
		0.100	13.41	0.500	16.57

R^2^: correlation coefficient; LOQ: Limit of Quantification (LOQ < MRL*); LOD: Limit of detection; ±SD: Standard deviation; RSD: Relative standard deviation; values given in brackets, are the standard acceptance criteria as per SANTE, 2017 [26]; *Acetamiprid MRL-0.2 mg/kg in okra and ethion MRL-5 mg/kg in green chili.

**Table 2:** Dietary risk assessment of acetamiprid in okra for different groups of Indian consumers.

Group	Particulars with Age	Food Consumption (g/day)	Body Weight (kg)	Dietary Risk Assessment						
				Acetamiprid @20 g a.i./ha (RD)						
				RQ ^a^	RQ ^b^	RQ ^c^	RQ ^d^	RQ ^e^	RQ ^f^	RQ ^g^
Children	1–3 years	50	12.90	0.1	0.1	0.1	0.1	0.1	0.1	0.1
	4–6 years	100	18.00	0.1	0.1	0.1	0.1	0.1	0.1	0.1
	7–9 years	100	25.10	0.1	0.1	0.1	0.1	0.1	0.1	0.1
Boys	10–12 years	200	34.30	0.1	0.1	0.1	0.1	0.1	0.1	0.1
Girls	10–12 years	200	35.00	0.1	0.1	0.1	0.1	0.1	0.1	0.1
Boys	13–15 years	200	47.60	0.1	0.1	0.1	0.1	0.1	0.1	0.1
Girls	13–15 years	200	46.60	0.1	0.1	0.1	0.1	0.1	0.1	0.1
Boys	16–18 years	200	55.40	0.1	0.1	0.1	0.0	0.0	0.0	0.1
Girls	16–18 years	200	52.10	0.1	0.1	0.1	0.1	0.1	0.1	0.1
Man	Sedentary work	200	60.00	0.1	0.1	0.1	0.0	0.0	0.0	0.1
	Moderate work			0.1	0.1	0.1	0.0	0.0	0.0	0.1
	Heavy work			0.1	0.1	0.1	0.0	0.0	0.0	0.1
Woman	Sedentary work	200	55.00	0.1	0.1	0.1	0.0	0.0	0.0	0.1
	Moderate work			0.1	0.1	0.1	0.0	0.0	0.0	0.1
	Heavy work			0.1	0.1	0.1	0.0	0.0	0.0	0.1

RD: Recommended dose; RQ: Risk quotient; a: W0T0-Treated unwashed control (non-ozonated); b: W1T1-washing with tap water for 3 min; c: W1T2-washing with tap water for 8 min; d: W1T3-washing with tap water for 10 min; e: W2T1-washing with ozonized water for 3 min; f: W2T2-washing with ozonized water for 8 min; g: W2T3-washing with ozonized water for 10 min.

**Table 3:** Dietary risk assessment of ethion in green chili for different groups of Indian consumers.

Group	Particulars with Age	Food Consumption (g/day)	Body Weight (kg)	Dietary Risk Assessment						
				Ethion @1000 g a.i./ha (RD)						
				RQ ^a^	RQ ^b^	RQ ^c^	RQ ^d^	RQ ^e^	RQ ^f^	RQ ^g^
Children	1–3 years	50	12.90	0.0	0.0	0.0	0.0	0.0	0.0	0.0
	4–6 years	100	18.00	0.0	0.0	0.0	0.0	0.0	0.0	0.0
	7–9 years	100	25.10	0.0	0.0	0.0	0.0	0.0	0.0	0.0
Boys	10–12 years	200	34.30	0.0	0.0	0.0	0.0	0.0	0.0	0.0
Girls	10–12 years	200	35.00	0.0	0.0	0.0	0.0	0.0	0.0	0.0
Boys	13–15 years	200	47.60	0.0	0.0	0.0	0.0	0.0	0.0	0.0
Girls	13–15 years	200	46.60	0.0	0.0	0.0	0.0	0.0	0.0	0.0
Boys	16–18 years	200	55.40	0.0	0.0	0.0	0.0	0.0	0.0	0.0
Girls	16–18 years	200	52.10	0.0	0.0	0.0	0.0	0.0	0.0	0.0
Man	Sedentary work	200	60.00	0.0	0.0	0.0	0.0	0.0	0.0	0.0
	Moderate work			0.0	0.0	0.0	0.0	0.0	0.0	0.0
	Heavy work			0.0	0.0	0.0	0.0	0.0	0.0	0.0
Woman	Sedentary work	200	55.00	0.0	0.0	0.0	0.0	0.0	0.0	0.0
	Moderate work			0.0	0.0	0.0	0.0	0.0	0.0	0.0
	Heavy work			0.0	0.0	0.0	0.0	0.0	0.0	0.0

RD: Recommended dose; RQ: Risk quotient; a: W0T0-treated unwashed control (non-ozonated); b: W1T1-washing with tap water for 3 min; c: W1T2-washing with tap water for 8 min; d: W1T3-washing with tap water for 10 min; e: W2T1-washing with ozonized water for 3 min; f: W2T2-washing with ozonized water for 8 min; g: W2T3-washing with ozonized water for 10 min.

## Data Availability

Not applicable.
